# Consumption of 100% Pure Fruit Juice and Dietary Quality in French Adults: Analysis of a Nationally Representative Survey in the Context of the WHO Recommended Limitation of Free Sugars

**DOI:** 10.3390/nu10040459

**Published:** 2018-04-07

**Authors:** France Bellisle, Pascale Hébel, Alice Fourniret, Eléna Sauvage

**Affiliations:** 1 Nutritional Epidemiology Research Team, Université Paris 13, INSERM (U1153), INRA (U1125), Cnam, 93017 Bobigny, France; 2 CREDOC (Centre de Recherche pour l’Etude et l’Observation des Conditions de Vie), 142 rue du Chevaleret, 75013 Paris, France; hebel@credoc.fr (P.H.); fourniret@credoc.fr (A.F.); sauvage@credoc.fr (E.S.)

**Keywords:** fruit juice, BMI, diet quality, free sugar, sugar-containing beverages, WHO recommendation

## Abstract

Sugar-containing beverages are often seen as a negative influence on diet quality and body weight control. The present study examines the consumption of 100% fruit juice (FJ) based on a seven-day dietary survey in a representative sample of French adults (*n* = 1607). About a half of the participants (44%) consumed FJ, most often at breakfast time (60%). Average intake in FJ consumers was 115.6 ± 4.0 mL/day (46.3 ± 1.7 kcal/day). Prevalence of consumption increased with education and income and decreased with age, but no association was observed with body mass index (BMI), physical activity, or smoking. In consumers, FJ brought 2% daily energy and contributed larger proportions of vitamins (B1 7%, B2 3%, B5 5%, B6 6%, B9 10%, C 32%, E 9%, beta-carotene 5%), minerals (magnesium 4%, potassium 7%), and free sugars (19%). FJ consumers ingested more whole fruits, vegetables, and many other foods than non-consumers did. Free sugars represented 11.2% of the daily energy in FJ consumers versus 8.6% in non-consumers. This cross-sectional survey reveals that FJ contributes to diet quality without association with excess body weight. These observations should be confirmed in longitudinal studies. They support the view that contribution to diet quality should be specifically recognized in the context of the World Health Organization (WHO) recommended decrease of free sugar intake.

## 1. Introduction

The World Health Organization (WHO) recommends that the daily intake of “free sugars” be limited to less than 10% of total energy intake for body weight control purposes [1]. The WHO defines free sugars as all monosaccharides and disaccharides added to foods by the manufacturer, cook, or consumer, plus sugars naturally present in honey, syrups, fruit juices, and fruit juice concentrates. The intake of sugar reported in the US [2] and many European countries [3] exceeds the WHO recommendation, which can represent a significant challenge for many consumers.

The consumption of sugar is frequently blamed as a determining cause of obesity, particularly in the form of sugar-containing beverages [4,5]. Several countries around the world have adopted selective taxes in order to decrease their consumption, such as France [6] and Mexico [7]. In addition to adding calories to the diet, sugar-containing beverages are also suspected of displacing the intake of other valuable foods (such as whole fruits) or beverages (milk) in the diet [8,9].

It remains unclear whether all sugar-containing beverages have the same impact on the quality of the diet and body weight control [10]. Among sugar containing beverages, some contain mainly added sugar (sodas) while others, such as 100% pure fruit juice (FJ), contribute nutrients along with sugar and calories. Many reviews have addressed the specific contribution of FJ to diet quality and weight control in children and adults. Hyson [11] reviewed observational and intervention studies examining consumption of pure fruit juice (PJ) and various aspects of health, including cancer, cardiovascular disease, cognition, hypertension, urinary tract infections, and body weight control. This review, spanning about two decades of research from around the world, concluded that PJ contains bioactive components with potential to affect human health positively, notably in terms of antioxidant and anti-inflammatory properties, improved lipid metabolism, cancer processes, and possibly body weight control, while insisting that more long-term clinical trials are needed to demonstrate clear outcomes and relevant mechanisms. The absence of association generally reported between PJ consumption and adverse outcomes in terms of body weight in adults or children suggests that dietary compensation or other mechanisms associated with components in PJ might account for the lack of weight gain, even in protocols where PJ provided additional calories. Similar observations and conclusions were reported in reviews examining the contribution of pure citrus juice to diet quality and weight status [12,13]: in addition to a substantial contribution to the intake of many nutrients, consumption of citrus juice appears not to be associated with body weight in children, while a limited number of epidemiological studies support an inverse association between the intake of orange juice and anthropometric measures in adults. A recent meta-analysis suggests that FJ contributes positively to one’s diet and could help satisfy fruit requirements in adults and children [14].

The present report is based on a recent nationally representative survey of the French population, the “Comportements et Consommations Alimentaires en France” (CCAF) study conducted in 2016 by the Centre de Recherche pour l’Etude et l’Observation des Conditions de Vie (Research Centre for the Observation of Life Conditions, CREDOC). Its goal is to examine FJ consumption and its nutritional and health correlates, particularly in terms of quality of diet (intake of nutrients and potential displacement of specific foods such as whole fruits and dairy) and body weight status.

## 2. Methods

### 2.1. Population

In France, CCAF surveys are periodically conducted in representative samples of the French population (children and adults). They constitute a database that can be exploited for the examination of specific aspects of nutrition [15,16]. The most recent CCAF survey was carried out between October 2015 and July 2016 in a nationally representative sample of 1288 households, in which all individuals over 3 years of age were interviewed, plus an extra national sample of individuals aged 3–19 years. Data was weighted on individuals 18 years old and over to account for this extra sample. Age, socio-economic status (based on occupation of head of household as classified by the National Institute of Statistics), geographical region, town size, and household size were taken into consideration in the quota sampling method. The general methodology of CCAF surveys has been published previously [17]. The total sample of the 2015–2016 study included 1607 adults (aged 21 years and older), 1164 children, and 318 adolescents. The present article will report data obtained in adults.

For each participant, self-reported height, weight, time spent on physical activity and sedentary behavior (screen watching), and dieting (for medical, religious, weight control, or any other reasons), as well as socio-demographic data (education, income, profession, composition of household, etc.), were recorded in face-to-face interviews. The participants completed a seven-day food intake survey either in paper (54.7%) or online (45.3%) formats. Paper versus online reports showed no significant differences in terms of energy intake or of consumption of most food categories, macro- and micronutrients. 

The energy intake reported by the participants was compared with the estimated energy requirements according to Schofield’s equation [18]. Under-reporters were excluded (declared energy intake ≤0.95 times the metabolic rate). This cut off value was chosen on the basis of Black’s practical guide [19] that proposed a lower 95% CL of 1.05 for moderately active individuals. Since the present population reported low levels of physical activity relative to previous CCAF surveys, it was arbitrarily decided to use a slightly lower cut-off value. From the total sample of the CCAF 2016 study, 1607 participants (887 men, 720 women) were included in the study, after exclusion of under-reporters (*n* = 652). There were no differences in gender distribution or education level between the included and excluded participants. The proportion of overweight and obese individuals among the excluded participants was greater than among the included participants (*X*^2^ text; *p* < 0.0001). 

The CCAF study was approved by the institutional review board for research in human subjects. All participants provided informed consent.

### 2.2. Dietary Intake Data

Dietary intake was assessed from a seven-day food intake record for all household members. The participants reported the types and amounts of all foods and beverages consumed. In order to facilitate portion reporting, the participants were provided with the validated SUVIMAX portion size atlas [20] showing various common foods and beverages in different portion sizes. 

The energy and nutrient contents of foods and drinks were obtained from the CIQUAL French Food Composition Table [21] updated in 2016. Thirty-eight mutually exclusive groups of foods/beverages were considered: bread & biscottes, cakes & pies, cheese, condiments, dried fruits, eggs & egg dishes, fats, fish & seafood, fruits, fruit desserts (sugar added), grains, legumes, meat, offals, pasta, pastries, pizzas & quiches, potatoes, poultry-game, processed meats, ready to eat breakfast cereals, ready-to-eat dishes, rice & cooked breakfast cereals, salty crackers & biscuits, sandwiches, sauces & salad dressings, soups, sugar & sweets, sweet crackers & biscuits, vegetables, viennoiseries, yoghurts & fresh dairy, alcoholic beverages, hot beverages, juice & nectars, milk, sodas, and water. PJ consumption was extracted from the juice & nectars group. Only 100% pure juice was considered for constituting “consumers” versus “non-consumers” groups, excluding “nectars” (fruit drinks plus sugar). The 100% pure juice category included juices from all fruits, either home-made or from commercial brand (no sugar added; either whole product or reconstituted from concentrate). 

Intakes were computed for the whole day and for individual eating occasions (meals and snacks). The circumstances of intake were reported by the participants, including time of day, day of the week, location, and context of consumption. The contents in added sugars of the CIQUAL foods and beverages were estimated using the systematic methodology proposed by Louie et al. [22] in which “added sugars” are defined as refined sugars added during cooking or manufacturing, in agreement with the USDA definition. 

### 2.3. Assessment of Daily Screen Watching Time and Physical Activity

The time spent watching various screens (television, computer, video games, etc.) as well as the time spent on various physical activities (household activities, gardening, sports, etc.) were reported by the participant. For physical activity, two levels were arbitrarily defined (low = less than 2 h/day; high = 2 h/day or more), in accordance with previous CREDOC studies. Daily screen watching was used as a proxy for sedentary behavior. Two levels were arbitrarily defined for screen watching time (low = less than 2 h/day; high = 2 h/day or more). 

### 2.4. Determination of Body Weight Status

BMI (Wt/Ht^2^) was computed from reported height and weight. Values between 18.5 and 25 kg/m^2^ were considered to represent normal body adiposity status. Overweight and obesity were defined as BMI values ≥ 25 and 30 kg/m^2^, respectively. Underweight corresponded to BMI values < 18.5 kg/m^2^.

### 2.5. Statistical Analyses

The SAS 9.4 software (SAS Institute, Inc., Cary, NY, USA) was used for statistical analyses and for database management. Differences between proportions were tested using *X*^2^ tests. Differences in quantitative variables (such as intakes) were tested using the linear model (PROC ANOVA) one-way ANOVAS. For continuous variables, Student’s t tests were used. Supplementary analyses (ANCOVAs) were performed in order to examine the relationship between weight/BMI as continuous variables, adjusted for age and sex, and consumption of FJ. The confidence level for calculated confidence intervals was 95%. The statistical significance level was set at *p* < 0.05. Means ± standard errors (SEM) are presented in the text and tables.

## 3. Results

### 3.1. Intake of FJ According to Age, Gender, and Socio-Economic Status (Table 1)

In the whole sample, 44% of respondents reported consuming FJ at least once in the seven-day food diary. In consumers only, the average daily intake was 115.6 ± 4.0 mL (46.3 ± 1.7 kcal) and the average weekly number of consumption events was 4.9. Pure orange juice represented over half of the total intake (54%). Almost all FJ consumption took place at home (86%). Half of the weekly intake occurred in the company of others. Breakfast was the main occasion of consumption (60%) followed by lunch (10%), dinner (8%), and morning, afternoon, or evening snacks.

Table 1 presents the distribution of consumers and non-consumers of FJ according to gender, age, and other individual and social characteristics. Higher proportions of consumers were found among women compared with men (*p* = 0.006) and among younger compared with older participants (*p* < 0.0001). Prevalence of consumption increased with education and income. Higher prevalence was found in the Paris area and in the south of the country than in the north. Family composition, food budget, daily screen time, physical activity level, smoking status, dieting, and BMI categories had no significant influence. Supplementary analyses of BMI and weight, as continuous variables with adjustment for age and sex, and FJ consumption confirmed non-significant associations (for BMI, F(1,1606) = 1.72, *p* = 0.19; for weight, F(1,1606) = 0.04, *p* = 0.835).

### 3.2. Food Options in the Diet of Consumers And Non-Consumers of FJ

**Figure 1:**
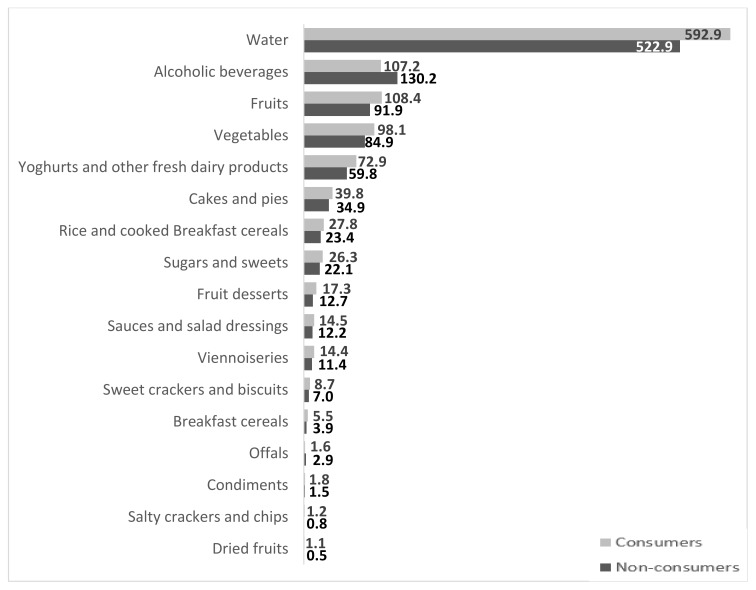
Significant differences (*p* < 0.05) in daily intake (g/day) of various food groups in consumers (light bars) versus non-consumers (dark bars) of FJ. A total of 37 mutually exclusive food groups were compared using a Student’s *t*-tests.

Figure 1 presents the significant differences in consumption of specific food groups, according to FJ consumption status. A total of 37 comparisons were made, according to the list of 38 food groups provided in the Methods section, minus the FJ group. Consumers of FJ also consumed significantly more water, fruits, vegetables, yogurts and other fresh dairy products, several types of sweet tasting foods (cakes, pies, fruit desserts, sweet crackers and biscuits, and dried fruits) and more sugar and sweets than did non-consumers. They consumed fewer alcoholic beverages. 

All significant differences in food choices appear in Figure 1. Comparisons of all other food groups, such as sodas (77.2 ± 5.8 vs. 77.7 ± 6.3 mL per day, respectively), showed no significant differences between FJ consumers and non-consumers.

### 3.3. Diet Composition and Quality in Consumers Versus Non-Consumers

The daily intake of energy and macro- and micronutrients was consistently higher in consumers of FJ than in non-consumers. Many significant differences appeared (Table 2).

Consumers of FJ reported a significantly higher daily energy intake than non-consumers (2029 ± 21 vs. 1941 ± 17 kcal). Their diet also brought more CHO (229 ± 2.7 vs. 213 ± 2.2 g), free sugars (57 ± 1 vs. 42 ± 1 g), fiber (20 ± 0.3 g vs. 19 ± 0.2 g), and fats (80 ± 1 g vs. 76 ± 1 g), but the differences in protein and starch intake did not reach significance. Free sugars (according to the WHO definition) represented 11.3% of daily energy in consumers versus 8.7% in non-consumers. Significantly higher intakes of many vitamins and minerals were observed in consumers of FJ, notably vitamins C and E, potassium, and magnesium. Table 2 also presents nutrient density (intake per 2000 kcal). The diet of FJ consumers (versus non-consumers) was significantly denser in CHO, simple and free sugars, many vitamins (B1, B6, B9, C, and E), beta-carotene, potassium, magnesium, and manganese. Conversely, it was less dense in starch, protein, cholesterol, B12, zinc, sodium, and phosphorous.

**Figure 2:**
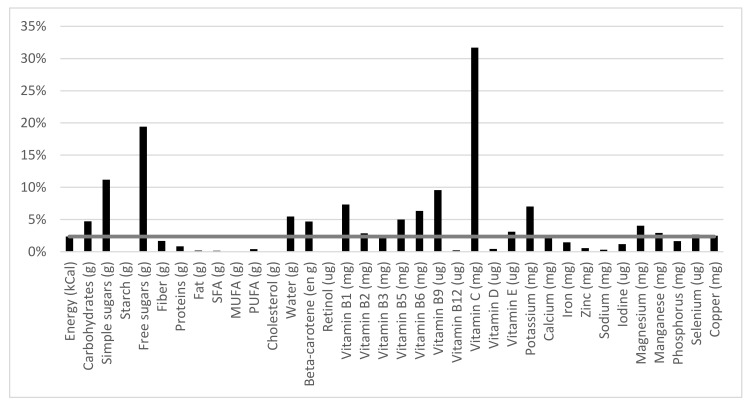
Contribution (%) of FJ to nutritional intakes in FJ consumers. The horizontal gray line projects the level of energy (2%) contributed by FJ.

FJ per se brought 2% of the daily energy in the diet of consumers. Figure 2 shows the contribution of FJ to the daily intake of various nutrients, relative to the 2% contribution to energy intake. FJ contributed in higher proportions to the daily intake of vitamins C (32%), B9 (10%), B6 (6%), B5 (5%), B2 (3%), and B1 (7%), beta-carotene (5%), manganese (3%), magnesium (4%), and potassium (7%). FJ contributed 19% of the daily free sugars (10.2 ± 0.4 g/day). By comparison, sugar-containing sodas brought 6.9 ± 0.1 g of free sugar per day in FJ consumers. 

## 4. Discussion

One hundred percent pure FJ is a beverage that contributes no added sugar but nevertheless contains free sugars. According to the WHO, the intake of free sugars should be limited to less than 10% of total daily energy for weight control purposes. Sugar-containing beverages are often specifically blamed for inducing passive energy over-consumption, facilitating weight gain, and displacing healthy foods and beverages from the diet [4]. Unlike many other popular sugar-containing beverages, however, FJ also contributes to diet quality and is associated with a number of positive health factors [11,12,13,14]. The present analysis of a recent nationally representative survey in French adults casts light on a number of nutritional and health correlates that can assist in the evaluation of the role of FJ in the contemporary diet. 

About half of French adults consume 100% pure FJ. The daily consumption is modest: 116 ± 4 mL per day, representing about 46 kcal. Of note, the daily consumption of sugar-containing sodas in French adults is even more modest: 77 mL/day. More women than men consume FJ and larger proportions of consumers are found in younger than older adults. Proportions of consumers increase with education level and income, although the food budget does not appear a significant factor. Many other aspects of lifestyle are unrelated to FJ consumption: the number of persons in the household (particularly the presence of children), daily time spent watching screens or doing physical activity, smoking status, dieting, and body weight status. 

The levels of FJ consumption reported in the CCAF 2016 sample agree with other reports. The recently published 3rd INCA study [23], a major nationally representative survey of the French population, using a different methodology (three 24 h recalls rather than a seven-day food diary, among other differences), reports 50.3% consumers who ingest 127 mL/day of fruit + vegetable juices. A previous CCAF survey conducted in 2010 [17] reported 52% consumers with a mean daily FJ intake of 115 mL. FJ consumption thus appears stable in French adults, both in terms of prevalence and amounts consumed.

In our sample, consumers of FJ consumed more of a large number of other foods and had a higher daily energy and nutrient intake than had non-consumers. The absence of association of FJ consumption with the BMI suggests that FJ consumers were more active individuals. The measures of physical activity and sedentary time (screen watching) did not reveal any significant difference between FJ consumers and non-consumers, however. It is possible that the assessment questions were too crude to detect actual differences. One obvious factor contributing to the significant differences in daily intake is age: FJ consumers were significantly younger than non-consumers. It is known that energy and sugar intake decreases with age in adults [2,23]. Some residual under-reporting cannot be ruled out.

The absence of a positive association between FJ consumption and BMI is consistent with other observations [14]. For example, Wang et al. [24] reported that, among the US adults participating in a cross-sectional NHANES survey, consumers of pure orange juice had a lower BMI, waist circumference, and body fat percentage than non-consumers. The level of juice intake in the American population (over 200 mL/day) was much higher than in the French sample, as was the level of soda intake (over 300 mL/day). As in the present survey, American consumers of juice reported lower alcohol consumption. The comparison between the NHANES and the CCAF data suggests that FJ consumption either is unrelated to BMI (in populations where FJ intake is modest) or shows an inverse association (in populations where FJ intake is higher). It may be that consumption of a food or beverage has to reach a certain level before its correlates can be detected, or that the NHANES survey was simply better able to detect a difference. 

FJ is one element in a complex, varied diet. Per se, FJ consumption contributed 2% of daily calories in FJ consumers and a higher proportion of the daily input of many vitamins and minerals. It also contributed free sugar. FJ consumers in our study reported higher intakes of many nutrients, both in absolute values and in nutrient density: CHO, simple and free sugars, many vitamins (notably C and E), and minerals. By contrast, their diet was significantly less dense in starch, protein, cholesterol, B12, zinc, sodium, and phosphorous. It could be that FJ consumption is one element in the diet of individuals who are attracted to sweet tasting options (whole fruits and pastries being other examples), while non-consumers could be mainly interested in savory items, accounting for their higher intakes of protein, cholesterol, vitamin B12, and sodium. 

Consistent with this hypothesis, daily energy from free sugar exceeded the 10% limit recommended by the WHO in FJ consumers (average 11.3%), while non-consumers had an average daily intake of free sugar amounting to 8.7% of total daily energy. FJ consumption contributed about 10 g of free sugar daily (19% of total daily free sugar intake). It is slightly more than the contribution from sodas (7 g/day). In French FJ consumers and non-consumers, however, the main sources of free sugar are not beverages but rather solid sweet foods such as candy, chocolate, cookies, and pastries [3]. In the context of the WHO recommendation, the issue of different sources of added sugars, with different contributions to nutritional status, calls for a discriminative analysis of positive and negative impacts [10].

Of note, the consumption of FJ in French adults was associated with a higher intake of whole fruits and vegetables as well as yogurts and other dairy products. These data rule out the displacement of other healthy foods in the diet of FJ consumers. 

In France, as well as in other areas of the world, the daily intake of fruit and vegetable is lower than recommendations (the classic “five-a-day” or about 400 g/day). An expert panel in North America recently suggested that FJ consumption could be one useful tool to satisfy America’s fruit gap [14]. This is consistent with the nutritional guidelines for the French population that allow one portion of 100% FJ to count as one of the five recommended servings. In our study, FJ consumers did actually consume more whole fruits and vegetables than non-consumers, although their total intake (about 206 g/day) remained below the recommended amount.

The strengths of the present study include the very demanding seven-day food record method used to obtain intake data and the large nationally representative sample. A potential limitation of the present design is the fact that participants were recruited as members of households, which may have decreased the variability of dietary responses. The exclusion of many overweight/obese under-reporters also possibly limits the sensitivity of the observations at the higher end of the body weight spectrum. The data are cross-sectional observations and thus do not allow any demonstration of causal effects. 

The analysis of physical activity and sedentary time is limited in the present study. Clearly screen watching time is a crude proxy for sedentary behaviors. Of note, the arbitrary 2 h/day cut-off for defining lower versus higher physical activity appeared to be close to the median and partitioned the whole sample in two sub-samples of approximately equal numbers. The self-declaration of body weight is particularly prone to under-declaration. However, in the present study, weights were reported during face-to-face interviews with highly trained staff, and possibly in the presence of other members of the household. Although this does not guarantee a highly precise report of body weight, it makes it likely that any gross misreporting would have been detected at this early stage. Of note, the overall proportions of overweight (31.9%) and obese (17.5%) individuals, including under-reporters, was very close to the proportions observed in the 2017 INCA 3 study (34% and 17%, respectively), in which body weight and height were measured rather than declared in a comparable representative population over the same time period [23].

Future studies should address longitudinal changes in participants’ food choices as they age. The very significant age effect raises the question of whether there is a “generation effect” that predicts that the younger respondents in the 2016 study will maintain their intake of FJ as they age, or whether they will progressively adopt the lower prevalence of consumption seen in older respondents of the present study. If longitudinal surveys reveal that FJ consumption is progressively replaced by intake of other foods as the individual ages, the ensuing changes in diet composition or quality should be assessed. Associations between FJ consumption and BMI and other health indicators should also be investigated in longitudinal cohort studies. 

## 5. Conclusions

This nationally representative survey shows a lack of association between FJ consumption and BMI, no sign that FJ displaces other healthy foods, and that FJ contributes to the intake of valuable nutrients as well as free sugar in the diet. In the context of the WHO recommendation to limit the consumption of free sugar, the issue of different sources of free sugars, particularly beverages, with different contributions to nutritional status, requires discriminative analysis of positive and negative impacts, preferably in longitudinal follow-up studies. 

## Figures and Tables

**Table 1:** Characteristics of consumers and non-consumers of 100% pure fruit juice (FJ).

		Non-Consumers		Consumers		
			Distribution		Distribution	*X*²
		Weighted *N*	%	Weighted *N*	%	*p* Value
Gender	Man	497	49.9%	335	43.4%	0.006
	Woman	499	50.1%	438	56.6%	
Age	21–24 years	44	4.5%	60	7.7%	0.000
	25–34 years	132	13.3%	159	20.6%	
	35–44 years	176	17.7%	117	15.1%	
	45–54 years	194	19.5%	144	18.6%	
	55–64 years	187	18.8%	122	15.7%	
	65 years and over	262	26.3%	172	22.2%	
Education	No diploma	96	9.7%	44	5.7%	0.000
	Less than high school degree	444	44.9%	269	35.0%	
	Vocational education	90	9.1%	86	11.2%	
	High school	106	10.7%	85	11.0%	
	Undergraduate	114	11.5%	110	14.3%	
	Graduate	140	14.1%	176	22.8%	
	Missing answer	6		2		
Annual household income						
	Less than 9,909 €	44	4.9%	31	4.6%	0.004
	From 9,909 € to 12,958 €	61	6.9%	38	5.6%	
	From 12,958 € to 18,294 €	137	15.5%	80	11.9%	
	From 18,294 € to 30,490 €	323	36.5%	217	32.2%	
	More than 30 490 €	320	36.2%	308	45.7%	
	Missing answer	111		99		
Number of people in household						
	1	230	23.1%	172	22.3%	0.290
	2	409	41.1%	320	41.3%	
	3	133	13.4%	123	15.9%	
	4	141	14.1%	111	14.3%	
	5 or more	83	8.4%	48	6.2%	
Profession	Farm owner, craftsman, storekeeper, business owner, etc.	128	12.9%	56	7.3%	0.000
	Manager, executive, intellectual profession, professionals, etc.	140	14.1%	160	20.7%	
	Middle-level profession	211	21.2%	201	26.1%	
	Employees	173	17.4%	149	19.2%	
	Workers	301	30.2%	171	22.1%	
	Unemployed/never employed	43	4.3%	35	4.6%	
Region *	Paris area	163	16.3%	151	19.6%	0.032
	Northern France	485	48.7%	330	42.8%	
	Southern France	348	34.9%	291	37.7%	
Area size	<2000 hab.	260	26.1%	170	22.0%	0.037
	2000–20,000 hab.	169	17%	125	16.1%	
	20,000–100,000 hab.	125	12.6%	98	12.7%	
	>100,000 hab.	313	31.4%	242	31.3%	
	Paris urban area	129	12.9%	138	17.9%	
Family type	One person	230	23.1%	172	22.3%	0.481
	Couple without children	356	35.8%	270	35.0%	
	Couple with children	315	31.7%	252	32.6%	
	Single-parent family	70	7%	66	8.5%	
	Other type	25	2.5%	12	1.5%	
Food budget per person in the household						
	0–55 € per week	332	33.4%	235	30.5%	0.427
	55–90 € per week	362	36.3%	293	37.9%	
	More than 90 € per week	302	30.3%	245	31.7%	
Daily time of screen watching						
	Low	392	39.3%	287	37.1%	0.334
	High	604	60.7%	486	62.9%	
Physical activity	Low	515	51.7%	432	55.9%	0.077
	High	481	48.3%	341	44.1%	
Smoking	Non-smoker	304	69.5%	547	70.9%	0.770
	Smoker	691	30.5%	225	29.1%	
	Missing answer	1		1		
Dieting	Yes	153	15.4%	134	17.4%	0.294
	No	842	84.6%	639	82.6%	
Body Mass Index	Underweight	24	3.5%	27	2.4%	0.453
	Normal	528	54.4%	420	53.0%	
	Overweight	302	27.9%	215	30.3%	
	Obese	141	14.3%	111	14.2%	

* Northern France includes Alsace, Basse-Normandie, Bourgogne, Bretagne, Centre-Val de Loire, Champagne-Ardenne, Franche-Comté, Haute-Normandie, Lorraine, Nord-Pas-de-Calais, Pays de la Loire, Picardie, and Poitou-Charentes; Southern France includes Aquitaine, Auvergne, Corsica, Languedoc-Roussillon, Limousin, Midi-Pyrénées, and Provence-Alpes-Côte d’Azur. Paris area includes Ile-de-France.

**Table 2:** Daily energy and nutrient intake in FJ consumers (weighted *n* = 773) and non-consumers (weighted *n* = 996).

	Total Energy and Nutrient Intakes					Nutrient Intakes per 2000 kcal				
	Non-Consumers		Consumers		Student’s *T* Test	Non-Consumers		Consumers		Student’s *T* Test
Nutrient	Mean	SEM	Mean	SEM	*p* Value	Mean	SEM	Mean	SEM	*p* Value
Energy (kCal)	1941.4	16.7	2028.8	20.5	0.001	2000.0	0.0	2000.0	0.0	
CHO (g)	212.5	2.2	229.3	2.7	<0.0001	218.7	1.2	225.7	1.2	<0.0001
Simple sugars (g)	76.3	1.2	94.4	1.4	<0.0001	78.5	1.0	93.6	1.0	0.0001
Starch (g)	113.7	1.4	113	1.8	0.776	117.0	1.0	110.4	1.0	<0.0001
Free sugars (g)	42.3	1	57.2	1.2	<0.0001	43.0	0.9	56.2	0.9	0.0001
Fiber (g)	18.6	0.2	19.8	0.3	<0.0001	19.4	0.2	19.8	0.2	0.147
Proteins (g)	82.3	0.8	81.3	0.9	0.406	85.6	0.5	81.0	0.6	0.0001
Total fats (g)	76.2	0.8	80.3	1	0.001	78.6	0.5	79.0	0.5	0.473
Saturated fats (g)	31.1	0.4	32.3	0.5	0.038	31.9	0.3	31.7	0.3	0.542
Monoinsaturated fats (g)	26.2	0.3	27.8	0.4	0.001	27.1	0.2	27.5	0.2	0.195
Polyinsaturated fats (g)	9.4	0.1	9.9	0.1	0.006	9.7	0.1	9.8	0.1	0.335
Cholesterol (mg)	309.7	4.1	304	4.8	0.369	321.7	3.6	300.9	3.9	<0.0001
Water (mL)	1862.6	18.7	2020.8	23.9	<0.0001	1983.8	21.2	2067.1	25.6	0.012
Beta-carotene (µg)	2314.2	75.7	2799.9	92	0.001	2484.5	88.6	2849.9	85.9	0.004
Retinol (µg)	556.1	28.4	502.6	27.4	0.183	561.7	25.6	486.6	23.5	0.035
Vitamin B1 (µg)	1.1	0	1.2	0	<0.0001	1.1	0.0	1.2	0.0	<0.0001
Vitamin B2 (µg)	1.5	0	1.6	0	0.039	1.6	0.0	1.6	0.0	0.977
Vitamin B3 (µg)	17.8	0.3	18.5	0.3	0.065	18.6	0.2	18.5	0.3	0.860
Vitamin B5 (µg)	4.4	0.1	4.6	0.1	0.002	4.5	0.0	4.6	0.0	0.232
Vitamin B6 (µg)	1.5	0	1.6	0	<0.0001	1.55	0.0	1.63	0.0	<0.0001
Vitamin B9 (µg)	253.3	3.1	298.1	3.8	<0.0001	266.2	3.0	299.1	3.2	0.0001
Vitamin B12 (µg)	5.1	0.1	4.6	0.1	0.017	5.3	0.1	4.6	0.1	<0.0001
Vitamin C (µg)	64.3	1.6	100.6	2	<0.0001	68.6	1.7	102.2	2.0	0.0001
Vitamin D (µg)	2.5	0.1	2.6	0.1	0.198	2.7	0.1	2.7	0.1	0.972
Vitamin E (µg)	7.9	0.1	9	0.1	<0.0001	8.1	0.1	8.9	0.1	0.000
Potassium (mg)	2735.9	28	2946.2	29.3	<0.0001	2868.1	23.9	2962.8	23.3	0.005
Calcium (mg)	794.9	10.9	837.3	12.5	0.010	827.7	9.5	840.4	10.4	0.369
Iron (mg)	10	0.1	10.4	0.1	0.032	10.4	0.1	10.4	0.1	0.676
Zinc (mg)	8.9	0.1	8.8	0.1	0.778	9.2	0.1	8.8	0.1	<0.0001
Sodium (mg)	2933.1	31	2884.8	35.1	0.302	3036.0	20.4	2859.7	21.3	0.0001
Iodine (µg)	114.8	1.4	118.9	1.5	0.053	120.4	1.3	120.2	1.4	0.921
Magnesium (mg)	290.9	3.3	318.9	4.6	<0.0001	303.8	2.9	319.6	4.0	0.001
Manganese (mg)	2.5	0	2.8	0.1	<0.0001	2.6	0.0	2.9	0.1	0.002
Phosphorus (mg)	1158	11.3	1171	12.3	0.441	1201.2	7.2	1168.5	7.4	0.002
Selenium (µg)	96.2	1.2	99.1	1.4	0.103	101.8	1.2	100.9	1.4	0.637
Copper (mg)	1.4	0	1.4	0	0.917	1.5	0.0	1.4	0.0	0.176
